# Distinct liver sections exhibit sex-specific gene expression patterns in Lewis rats

**DOI:** 10.1038/s41598-025-17729-0

**Published:** 2025-09-01

**Authors:** Luisa Sophie Rajcsanyi, Simge Oral, Julia Borgardt, Florian Vondran, Oliver Beetz, Joanne Sambou, Selma Ugurel, Triinu Peters, Stefanie B. Flohé, Andrea Kindler-Röhrborn, Bettina Budeus, Arzu Oezcelik, Anke Hinney

**Affiliations:** 1 Section of Molecular Genetics in Mental Disorders, LVR-University Hospital Essen, University of Duisburg-Essen, Essen, Germany; 2 Institute of Sex- and Gender-Sensitive Medicine, University Hospital Essen, Essen, Germany; 3 Center for Translational Neuro- and Behavioural Sciences, University Hospital Essen, Essen, Germany; 4 Department of General, Visceral and Transplantation Surgery, University Hospital Essen, Essen, Germany; 5 ReMediES, Department of General, Visceral and Transplant Surgery, Hannover Medical School, Hannover, Germany; 6 Department of General, Visceral, Pediatric and Transplant Surgery, University Hospital RWTH Aachen, Aachen, Germany; 7 Department of Dermatology, University Hospital Essen, University of Duisburg- Essen, Essen, Germany; 8 Department of Dermatology, Bielefeld University, Medical School and University Medical Center OWL, Klinikum Bielefeld Rosenhöhe, Bielefeld, Germany; 9 Department of Trauma, Hand and Reconstructive Surgery, University Hospital Essen, University of Duisburg-Essen, Essen, Germany; 10 Genomics and Transcriptomics Facility, University Hospital Essen, Essen, Germany

**Keywords:** Genetics, Gene expression

## Abstract

Sex-specific differences in liver gene expression have previously been reported in humans and rodents. Clinically, female-to-male liver transplants are known to be associated with adverse post-transplantation outcomes. However, the underlying molecular mechanisms remain largely unknown. Sex-specific gene expression differences may be involved in the post-transplantation outcomes. Here, we analyse sex-specific differences in liver gene expression of Lewis rats on a genome-wide scale. In total, 543 genes exhibited a differential gene expression between male (*n* = 4) and female (*n* = 4) rats, with the largest difference found for the transcript ENSRNOG00000009273.7 (log_2_FC = 10.69, *p* < 2.2*10^− 308^). Genes downregulated (*n* = 272) in males were enriched for cholesterol homeostasis and late oestrogen response. We further analysed inter- and intra-sex gene expression differences in three individual liver sections to evaluate liver heterogeneity. Although several genes exhibited a sex-specific expression in all three liver sections (*n* = 240), distinct expression patterns within each individual section were determined. Variations between sections were even evident within the same sex with male liver sections revealing more differentially expressed genes (male *n* = 40, female *n* = 11). Consequently, studies investigating liver-specific gene expressions should consider this intrahepatic heterogeneity to avoid introducing potential biases. Subsequent studies ought to explore gene expression differences between the sexes pre- and post-transplantation, particularly regarding a female-to-male transplants.

**Supplementary Information:**

The online version contains supplementary material available at 10.1038/s41598-025-17729-0.

## Introduction

Sex-specific gene expression patterns in human, murine and rat tissues have previously been reported^1–4^. Particularly, cytochrome P450 genes exhibit distinct expressions between males and females^1–3^. In fact, *Cyp2c13* and *Cyp2c11* are more highly expressed in male rats, whereas *Cyp2c12* is generally upregulated in female animals^2,3^. These sex-specific expression patterns of cytochrome P450 genes are mainly driven by differences in plasma growth hormone secretion, which affects key transcription factors such as the signal transducer and activator of transcription 5b (STAT5B). This, in turn, modulates the activity of cytochrome P450 genes^5,6^.

Beyond the well-established sex-specific gene expression differences, biological differences between males and females display a significant clinical relevance, particularly in the context of liver transplantation outcomes. Studies in both humans and rats imply that post-transplantation outcomes are poorer when a female liver is transplanted into a male recipient than in any other combination^7–10^. Specifically, patients undergoing a female-to-male transplantation often experience shorter graft survival compared to those receiving sex-matched organs^11,12^. This effect appears to be particularly pronounced when the female donor was under 40 years of age and had no macrosteasosis^9,11^suggesting a putative role of hormonal factors. In contrast, male-to-female transplantations predominantly yield outcomes comparable to sex-matched combinations^12^. Thus, liver allocation models integrating sex-specific differences may improve the outcome of liver transplantation. However, in order to integrate sex differences effectively in the allocation model, the underlying mechanisms need to be fully elucidated. Already described sex-specific hepatic gene expression patterns may play a pivotal role in rendering these outcomes.

Previous studies on sex-specific differences in hepatic gene expressions have predominantly focused on small pre-defined subsets of genes, employing either quantitative polymerase chain reactions (qPCR)^3^ or microarray-based approaches^2,4^. Consequently, the present study aimed to provide a genome-wide coverage of sex-specific gene expressions by sequencing total RNA from liver samples of both male and female Lewis rats. To further assess the potential heterogeneity across different sections of the liver, three individual liver sections were analysed regarding intra- and inter-sex-specific variations in gene expression.

## Results

To comprehensively investigate sex-specific gene expression differences in rat liver on a genome-wide scale, we sequenced total hepatic RNA of male (*n* = 4) and female (*n* = 4) Lewis rats. The rats were approximately 12 weeks of age at the time of organ harvest. The livers were segmented in predefined anatomical sections corresponding to the right lateral lobe, the median lobe and the left lateral lobe. To study the heterogeneity of gene expression within the liver, the individual liver sections were analysed both, intra- and intersexually (see Fig. 7).

### Sex-specific gene expression differences across all liver sections

**Fig. 1:**
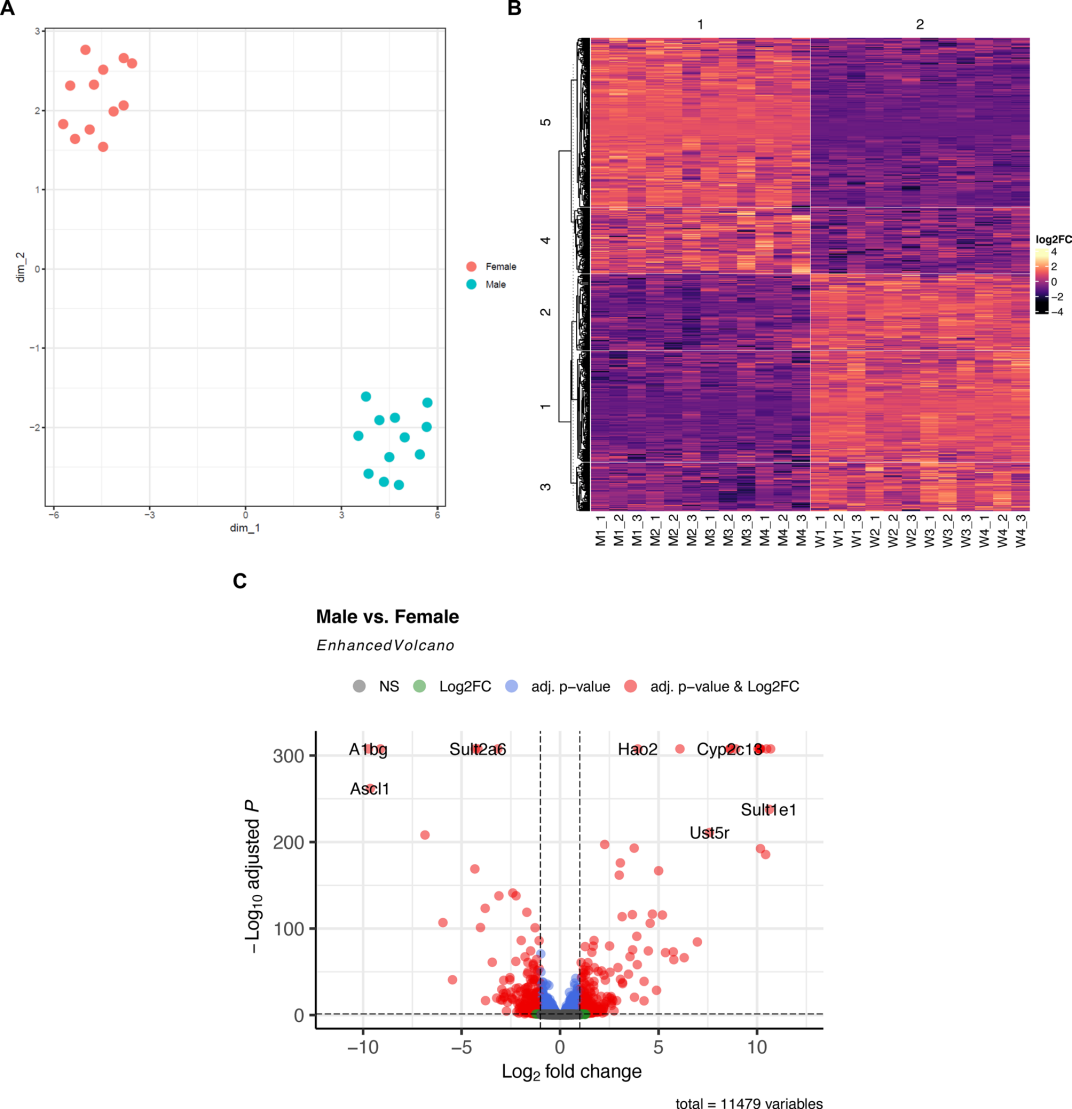
Results of the differential gene expression analysis across all liver sections of male and female rats. (**A**) The Uniform Manifold Approximation and Projection (UMAP) plot illustrates all analysed samples (*n* = 24) retrieved from three liver sections each of female (*n* = 4) and male (*n* = 4) Lewis rat. Red points indicate samples from female rats liver sections, while blue points represent male rat liver sections. (**B**) The heatmap visualizes the distinct and sex-specific gene expression patterns. Each row corresponds to a differentially expressed gene (p_adj_ < 0.05, *|*log_2_FC*|* > 1), while each column represents an individual rat liver sample. Lighter colour indicates higher gene expression in male than female liver sections (positive log_2_FC), whereas darker colours correspond to negative log_2_FC and a lower expression in male than in female rats. (**C**) The volcano plot illustrates differentially expressed genes between male and female livers across all individual sections. Following the initial filtering and exclusion of genes only expressed in one sample as well as genes with a mean expression below five, a total of 11,479 genes remained in the analysis. The 20 genes with the smallest p_adj_, for which a gene symbol is known, are labelled.

Initially, we examined gene expression differences between male and female rats across all liver sections. Uniform Manifold Approximation and Projection (UMAP) analysis revealed a distinct separation between samples of male and female rat livers (see Fig. 1A). In total, 543 genes demonstrated a differential expression between the sexes (p_adj_ < 0.05, |log_2_FC| > 1, mean |log_2_FC| = 1.92; max. |log_2_FC| = 10.69); see Fig. 1B and C, see Supplementary Table 1), with 272 (50.09%) being upregulated in male compared to female rats. The gene with the strongest upregulation (ENSRNOG00000009273.7, log_2_FC = 10.69, p_adj_ < 2.2*10^− 308^; see Supplementary Fig. 1A, see Supplementary Table 1) exhibited an ~ 1652-fold higher expression in males than females. In contrast, the gene with the strongest downregulation in male rats relative to females, *A1bg*, was approximately 849-fold lower expressed in males (ENSRNOG00000004692.7, log_2_FC = -9.73, p_adj_ < 2.2*10^− 308^; see Fig. 1C, see Supplementary Fig. 1B, see Supplementary Table 1).

**Fig. 2:**
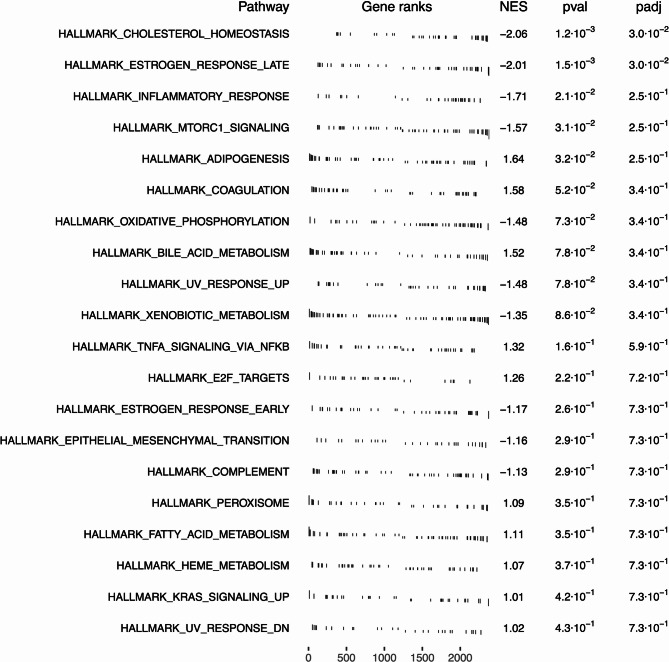
GSEA using the human ‘Hallmark’ gene set for differentially expressed genes between male and female rats across all liver sections. GSEA was performed using the R package ‘fgsea’^15^ to identify pathways that are enriched for differentially expressed genes between male and female rats across all liver sections (p_adj_ < 0.05, |log_2_FC| > 1). Here, the 20 most significantly enriched pathways are presented. Positive normalized enrichment scores (NES) indicate pathways with an enrichment of genes upregulated in males, while negative NES correspond to pathway enrichments of genes downregulated in males. Pathways are ranked according to their NES. Pathway enrichment was considered significant if p_adj_ < 0.05.

To explore functional implications of the identified differentially expressed genes, we performed a gene set enrichment analysis (GSEA) using well-annotated human gene set collections from the ‘Molecular Signature Database’^13,14^. Given that the most well-annotated gene sets are based on the human genome, GSEA was initially performed on homologous human genes corresponding to differentially expressed genes in rats. Analyses of the ‘Hallmark’ gene set revealed significant enrichments of genes downregulated in male rats within pathways related to cholesterol homeostasis (normalized enrichment score (NES) = -2.06, p_adj_ = 3.0*10^− 2^) and late oestrogen response (NES = -2.01, p_adj_ = 3.0*10^− 2^; see Fig. 2). Consistent with this, we observed enrichment of male downregulated genes in cholesterol biosynthesis and metabolism in the curated gene set ‘C2’ (see Supplementary Fig. 2), particularly in hepatocytes (NES = -2.16, p_adj_ = 4.4*10^− 2^) and enterocytes (NES = − 2.48, p_adj_ = 4.8*10^− 3^). Cell type-specific enrichments further indicated that downregulated genes in male rats were predominantly expressed in Kupffer cells (NES = − 2.14, p_adj_ = 3.5*10^− 2^) and Cajal-Retzius cells (NES = -2.16, p_adj_ = 2.2*10^− 2^; see Supplementary Fig. 5). Notably, no significant enrichment was observed in gene sets corresponding to regulatory targets (‘C3’, see Supplementary Fig. 3) or gene ontology terms (‘C5’, see Supplementary Fig. 4).

Yet, to uncover putatively enriched pathways in *Rattus norvegicus*, we subsequently performed a gene ontology (GO) enrichment analysis for genes exhibiting a sex-specific expression across all liver sections (p_adj_ < 0.05, |log_2_FC| > 1) using PANTHER with the *R. norvegicus* database^16^. Of the 271 genes upregulated in males relative to females, 167 (61.62%) were uniquely mapped by PANTHER, while 104 remained unmapped. Among the genes downregulated in males, 152 (55.88%) were uniquely mapped to a gene ID, while 120 again remained unmapped. GO enrichment analysis was exclusively performed on uniquely mapped gene IDs for ‘GO biological process’, ‘GO molecular function’ and ‘GO cellular component’ applying the Fisher’s exact test with a Bonferroni correction for multiple testing.

Upregulated genes in males were enriched in 19 biological processes (BP, p_adjusted_ < 0.05; see Supplementary Fig. 6A) with the most significant being ‘diterpenoid metabolic process’ (GO:0016101, fold enrichment (FE) = 11.98, p_adjusted_ = 1.88*10^− 2^), ‘monocarboxylic acid catabolic process’ (GO:0072329, FE = 11, p_adjusted_ = 1.3*10^− 3^), and ‘terpenoid metabolic process’ (GO:0006721, FE = 10.65, p_adjusted_ = 4.16*10^− 2^; see Supplementary Fig. 6A). Conversely, downregulated genes in males compared to females were overrepresented in weight BP (p_adjusted_ < 0.05; see Supplementary Fig. 7A), particularly in the ‘vitamin E metabolic process’ (GO:0042360, FE = 90.28, p_adjusted_ = 2.7*10^− 2^), ‘fat-soluble vitamin metabolic process’ (GO:0006775, FE = 20.33, p_adjusted_ = 4.23*10^− 2^) and ‘response to nutrients’ (GO:0007584, FE = 6.18, p_adjusted_ = 6.65*10^− 4^; see Supplementary Fig. 7A).

The overrepresentation in ‘GO molecular function’ revealed that genes upregulated in males were enriched for 12 molecular functions (MF, see Supplementary Fig. 6B). The greatest enrichment was determined for ‘androsterone dehydrogenase activity’ (GO:0047023, FE = 42.14, p_adjusted_ = 5.8*10^− 3^), ‘alcohol dehydrogenase [NAD(P)+] activity’ (GO:0018455, FE = 13.47, p_adjusted_ = 1.72*10^− 2^) and ‘monocarboxylic acid binding’ (GO:0033293, FE = 1.77, p_adjusted_ = 1.27*10^− 2^, see Supplementary Fig. 6B). In contrast, no enrichment in MF was observed for the set of genes downregulated in males compared to females (see Supplementary Fig. 7B).

Within the ‘GO cellular component’ (CC) annotation data set, exclusively genes downregulated in males were found to be enriched for the ‘endomembrane system’ (GO:0012505, FE = 1.85, p_adjusted_ = 6.96*10^− 3^, see Supplementary Figs. 6 C and 7 C).

### Inter-sex gene expression differences in individual liver sections

Subsequently, we proceeded to analyse sex-specific gene expression differences in individual liver sections in order to assess putative heterogeneity within the liver. UMAP of samples included in these analyses within the individual liver sections revealed a clear separation between male and female samples (see Supplementary Fig. 8).

**Fig. 3:**
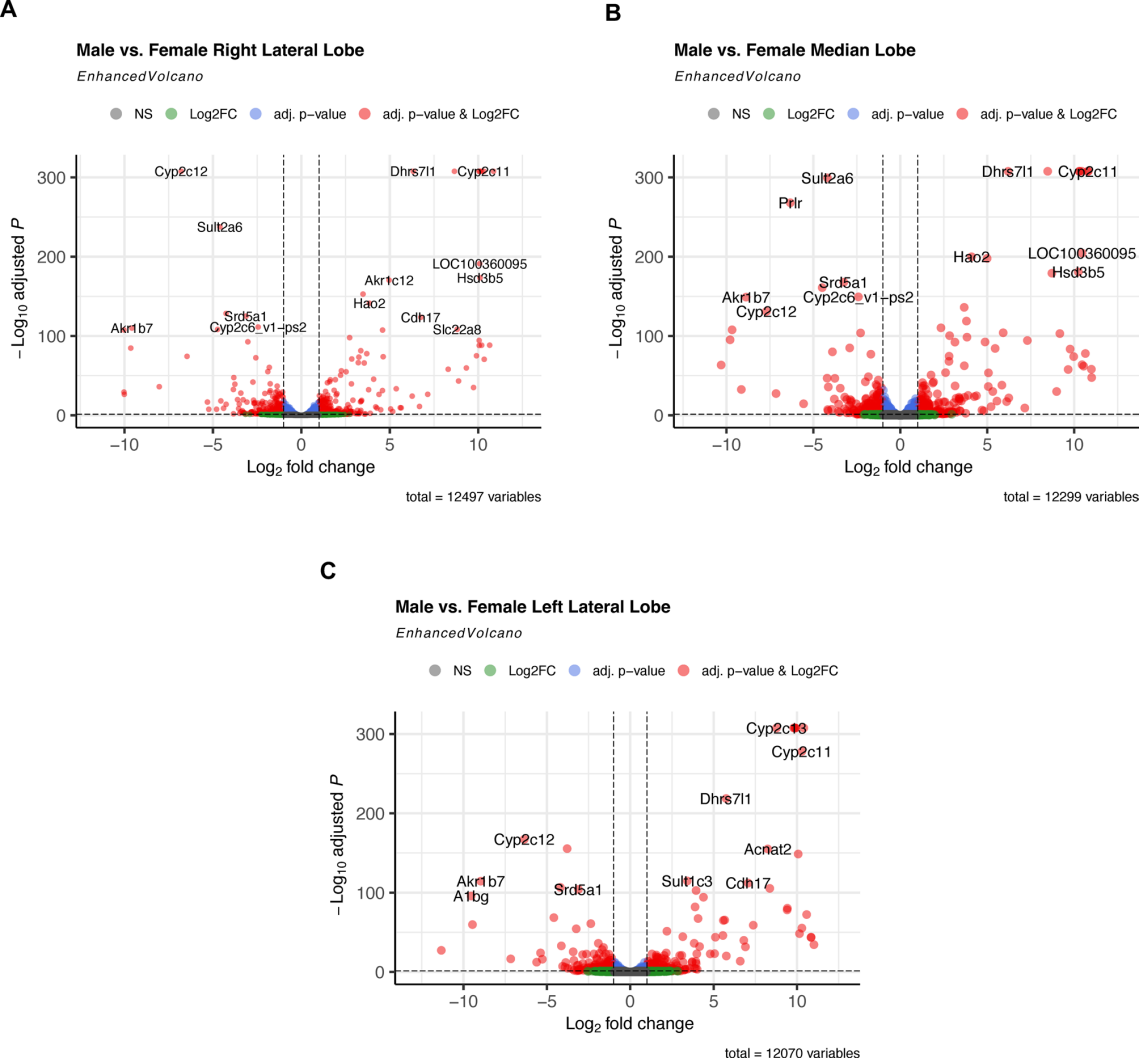
Volcano plot visualizing differentially expressed genes between male and female rats in distinct liver sections. The differential gene expression analyses were performed for liver samples of right lateral lobe (**A**), median lobe (**B**), and left lateral lobe (**C**) in male (*n* = 4) and female (*n* = 4) Lewis rats. All genes included in the analyses were expressed in at least one sample and exhibited a mean expression of at least five. The 20 genes with the smallest p_adj_, for which a gene symbol is known, were labelled.

Analysis of the right lateral lobe revealed 584 genes to have a sex-specific expression (p_adj_ < 0.05, |log_2_FC| > 1), with 303 being expressed at a higher level and 281 at a lower level in male compared to female rats (see Fig. 3A, see Supplementary Table 2). In accordance with our findings across all liver sections, *A1bg* was the most downregulated gene in male right lateral lobe relative to its expression in female right lateral lobe (log_2_FC = -10.03, p_adj_ = 2.23*10^–108^). *A1bg* was approximately 1046-fold lower expressed in the male right lateral lobe than in the female counterpart (see Supplementary Table 2).

254 genes were found to be upregulated, while 262 genes were downregulated in the male median lobe in comparison to corresponding female lobe (see Fig. 3B, see Supplementary Table 3). The most upregulated gene, ENSRNOG00000051081.1 (log_2_FC = 11.00, p_adj_ = 2.61*10^–48^), demonstrated a 2048-fold higher expression in males. Meanwhile, the most downregulated gene, ENSRNOG00000037549.3 (log_2_FC = -10.31, p_adj_ = 4.11*10^–64^), exhibited an approximately 1269-fold lower expression in male than in female rats’ median lobe (see Supplementary Table 3).

In the left lateral lobe, 650 genes exhibited a sex-specific expression (see Supplementary Table 4), with 342 being upregulated, while 308 were downregulated in males compared to females (see Fig. 3C). Similar to the findings in the median lobe, the most downregulated gene was ENSRNOG00000037549.3 (log_2_FC = -11.33, p_adj_ = 6.26*10^− 28^) with a 2574-fold attenuated expression in males than in females. The highest upregulation in males compared to females with an approximately 2062-fold upregulated gene expression, was detected for ENSRNOG00000046024.2 (log_2_FC = 11.01, p_adj_ = 4.05*10^− 35^).

**Fig. 4:**
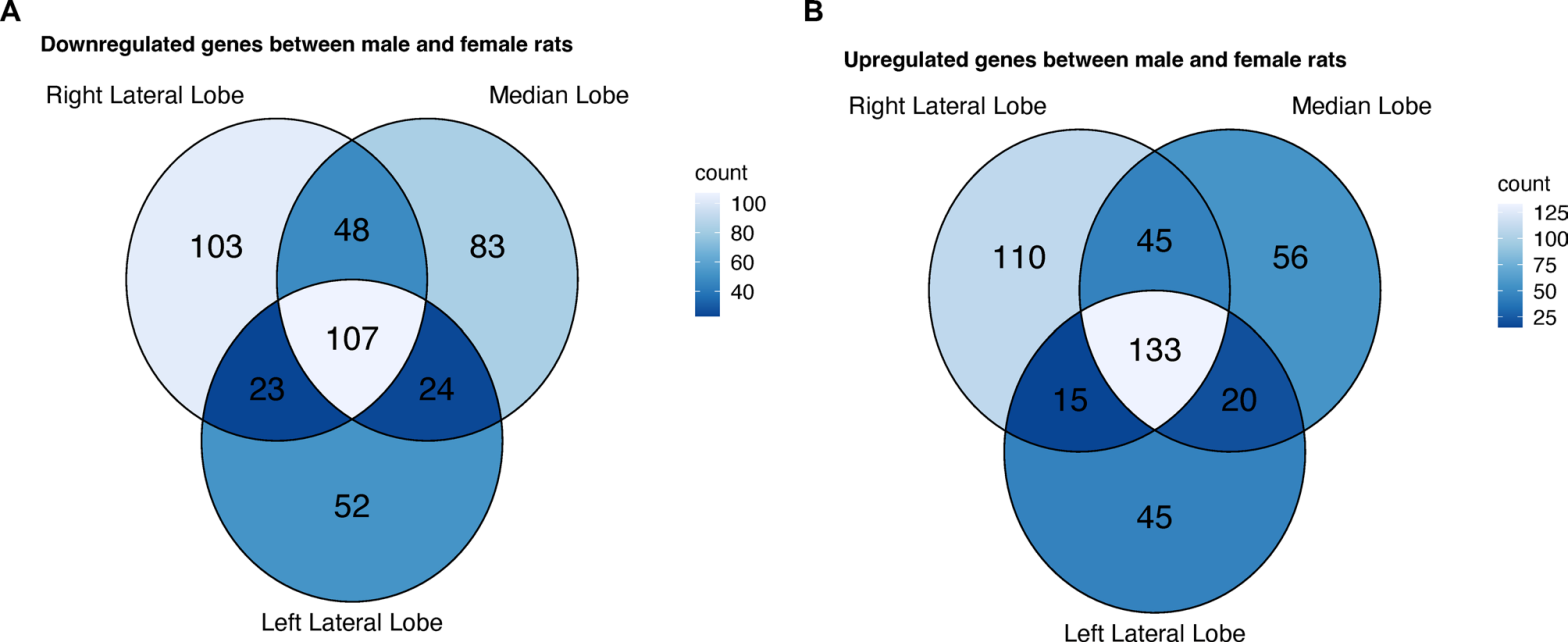
Venn diagram of genes with a sex-specific expression in the individual liver sections. The number of genes with a downregulation (**A**) as well as an upregulation (**B**) in male compared to female rats in each individual liver section are shown. The colour saturation indicates the number of genes detected to have a sex-specific expression in the respective liver section.

Despite discovering genes with sex-specific expressions (p_adj_ < 0.05, |log_2_FC| = 1) in all three individual liver sections, we concomitantly identified distinct sex-specific gene expression patterns within each individual section (see Fig. 4, see Supplementary Tables 5 and 6).

A total of 107 genes were consistently downregulated in males in all three individual liver sections (see Fig. 4A). Conversely, 103 genes demonstrated a downregulation exclusively in the male right lateral lobe, with ENSRNOG00000037911.2 exhibiting the greatest decrease in section specific expression in males (log_2_FC = − 8.03, p_adj_ = 9.22*10^− 37^). In the median lobe, 83 genes were specifically downregulated in males. Herein, ENSRNOG00000028092.2 demonstrated the most pronounced expression attenuation (log_2_FC = -7.15, p_adj_ = 3.94*10^− 28^). The left lateral lobe revealed 52 male-specific downregulated genes, with ENSRNOG00000010725.3 demonstrating the strongest section-specific downregulation in male rats relative to female animals (log_2_FC = -7.17, p_adj_ = 3.53*10^− 17^; see Supplementary Table 5).

Furthermore, several genes that were specifically downregulated in males were found to have an attenuated expression in two liver sections (see Fig. 4A). The most substantial overlap of genes downregulated in males was observed between the right lateral lobe and the median lobe (see Fig. 4A).

Likewise, a number of genes with an upregulation in males compared to females demonstrated section-specific expression patterns (see Fig. 4B). The highest number of upregulated genes with a section-specific differential expression between male and female rats was observed in the right lateral lobe (*n* = 110; see Fig. 4B). Here, the gene *Bhlha15* had the most pronounced gene expression increase in males compared to females (see Supplementary Table 6). In the median lobe, 56 genes exhibited a section-specific differential expression between the sexes (see Fig. 4B). The gene with the strongest upregulation in the median lobe of male rats in relation to female rats was ENSRNOG00000005639.7 (log_2_FC = 3.42, p_adj_ = 3.30*10^− 6^). A unique upregulation in the male left lateral lobe compared to the female equivalent was found for 45 genes (see Fig. 4B) with ENSRNOG00000046024.2 exhibiting the strongest expression increase in males compared to females (log_2_FC = 11.01, *p* = 4.05*10^− 35^, see Supplementary Table 6).

### Intra-sex differences in gene expression between liver sections

To elucidate a putative heterogeneity in gene expression between distinct liver sections that may introduce bias in studies exclusively analysing a single section, we subsequently investigated gene expression differences in individual liver sections within the same sex. The UMAP did consequently not allow a clear separation between the individual liver sections in either females (see Supplementary Fig. 9) or males (see Supplementary Fig. 10). Yet, a number of genes exhibited a differential expression between the liver sections in both female and male rats. Notably, these gene expression differences appeared to be less pronounced than in the analyses of sex-specific differences. Yet, in males more genes exhibited a differential expression between the sections than in female rats (see Supplementary Tables 7–12).

**Fig. 5:**
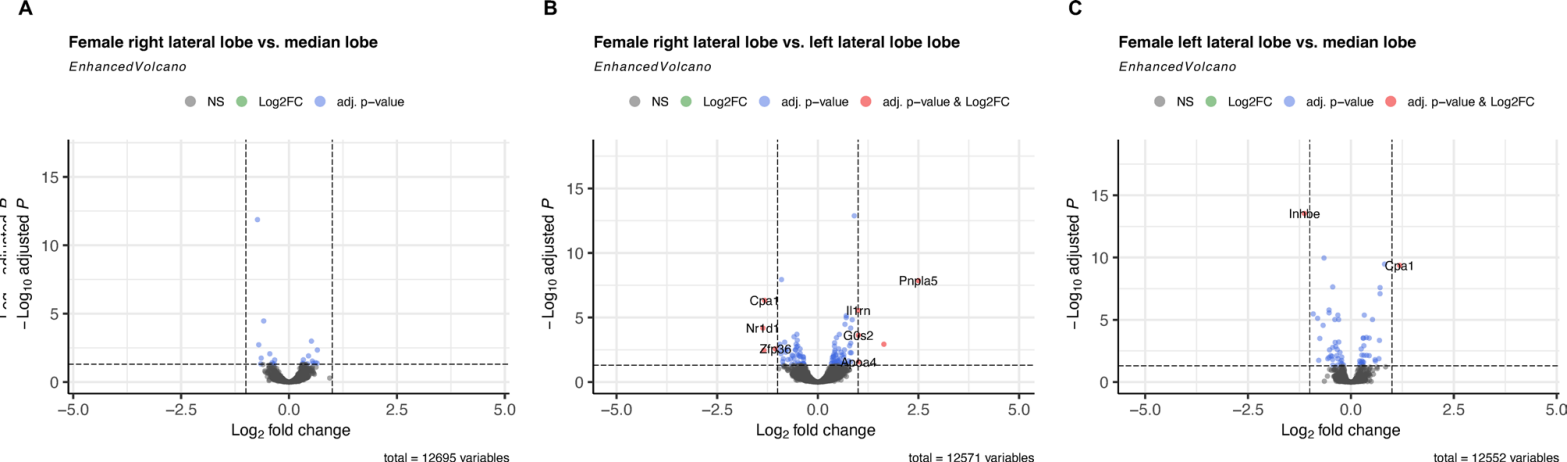
Volcano plot visualizing differentially expressed genes between lobular sections in female rats. The differential gene expression analyses were performed for liver samples of right lateral lobe vs. median lobe (**A**), right lateral lobe vs. left lateral lobe (**B**), and median lobe vs. left lateral lobe (**C**) in female (*n* = 4) Lewis rats. All genes included in the analyses were expressed in at least one sample and exhibited a mean expression of at least five. Genes labelled showed p_adj_ < 0.05 and |log_2_FC| > 1.

In female rats, nine genes showed a differential expression between the right and left lateral lobe (see Supplementary Table 8; see Fig. 5B), while two genes were either up- or downregulated in the median lobe in relation to the left lateral (see Supplementary Table 9, see Fig. 5C). The most upregulated gene in the female right lateral lobe in comparison to the left lateral lobe was *Pnpla5* (log_2_FC = 2.50, p_adj_ = 1.38*10^− 8^), whereas *Nr1d1* showed the most pronounced downregulation when comparing those liver sections (log_2_FC = -1.36, p_adj_ = 6.63*10^− 5^). In the median lobe of female rats, *Cpa1* was found to be significantly overexpressed in this section compared to the expression in the left lateral lobe (log_2_FC = 1.19, p_adj_ = 4.32*10^− 10^), while *Inhbe* was the most downregulated gene in the comparison of those liver sections in female rats (log_2_FC = -1.13, p_adj_ = 2.92*10^− 14^). However, no difference in gene expression was detected between the right lateral and median lobe (see Fig. 5A; see Supplementary Table 7).

**Fig. 6:**
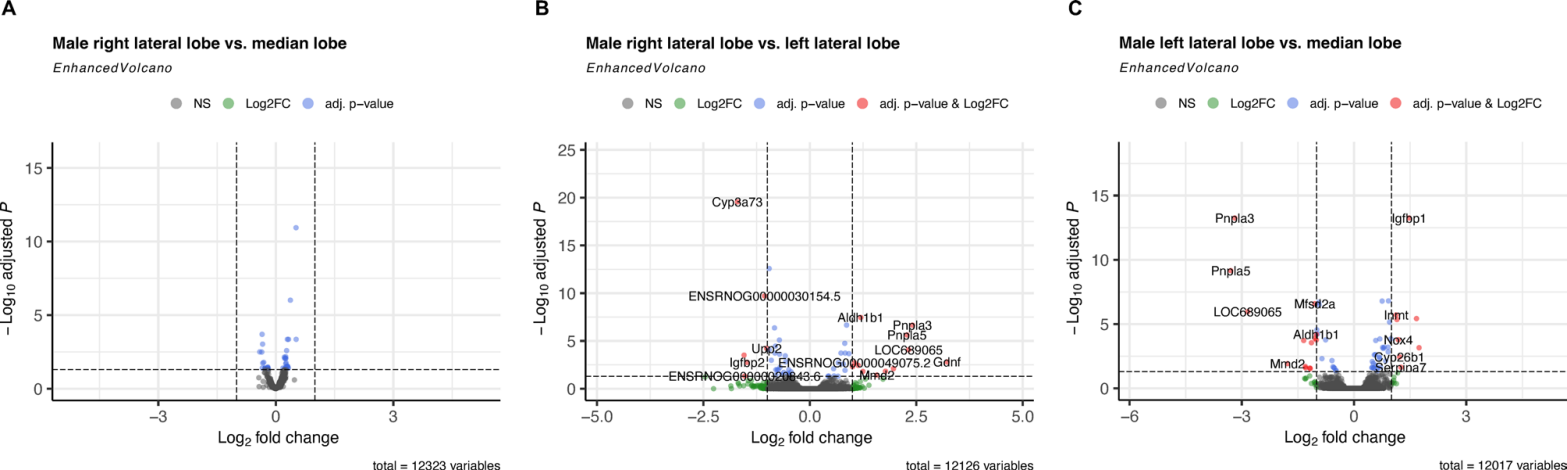
Volcano plot visualizing differentially expressed genes between lobular sections in male rats. The differential gene expression analyses were performed for liver samples of right lateral lobe vs. median lobe (**A**), right lateral lobe vs. left lateral lobe (**B**), and median lobe vs. left lateral lobe (**C**) in male (*n* = 4) Lewis rats. All genes included in the analyses were expressed in at least one sample and exhibited a mean expression of at least five. Genes labelled showed p_adj_ < 0.05 and |log_2_FC| > 1.

Among male rats, the highest number of genes with a differential expression between two liver sections were found when comparing the median and left lateral lobe (see Fig. 6C). Hereby, 22 genes had a differential gene expression between those sections with eight genes being up- and 14 being downregulated (see Supplementary Table 12). The most upregulated gene in the median lobe of male rats compared to the left lateral lobe was *Igfbp2* (log_2_FC = 1.74, p_adj_ = 6.94*10^− 4^). A differential expression between the left and right lateral lobe was observed for 18 genes (see Fig. 6B, see Supplementary Table 11) with *Gdnf* demonstrating the greatest upregulation in the right lateral lobe in relation to left lateral lobe of male rats (log_2_FC = 3.22, p_adj_ = 1.57*10^− 3^). The strongest downregulation, however, was determined for *Cyp3a73* (log_2_FC = -1.70, p_adj_ = 2.78*10^− 20^, see Supplementary Table 11). Notably, no gene expression differences were detected between the right lateral lobe and the median lobe of male rats (see Fig. 6A, see Supplementary Table 10).

## Discussion

Sex-specific gene expression patterns are well-established, particularly in rat livers. However, previous studies predominantly relied on pre-defined gene sets^2,3^ rather than achieving a genome-wide coverage. To address this limitation, we sequenced total hepatic RNA of male and female Lewis rats in order to allow an unbiased genome-wide investigation of sex-specific gene expression differences.

Our findings confirmed previously reported pronounced differences in gene expression of rat livers between males and females. A total of 543 genes revealed a sex-specific gene expression (p_adj_ < 0.05, |log_2_FC| > 1). Genes displaying a downregulation in male livers and correspondingly have a higher expression in female livers include *A1bg*, *Akr1b7*, as well as *Cyp2c12*, all of which have been previously reported to be sex-specific^2–4,17−20,6^. Notably, while *A1bg* and *Akr1b7*, were formerly exclusively detected in female or feminized male animals^17,18^our study revealed discernible expressions of both genes in male rats, albeit at levels substantially lower than those observed in female rats. This implies that the enhanced sensitivity of RNA-sequencing^21^as opposed to traditional methods such as Northern blots, may be crucial in the detection of low-abundance transcripts.

Additionally, we observed significant male-specific upregulations of *Cyp2c11* and *Cyp2c13*, with a substantial 1433-fold and 394-fold increase in male liver expression relative to female animals. These findings align with previous reports^2,3,19^demonstrating a 1700-fold and 1300-fold male-specific expression increase of *Cyp2c11* and *Cyp2c13*, respectively^3^. Similarly, *Cyp2c12* expression was markedly lower in males exhibiting a 116-fold downregulation, consistent with a previous study reporting a 200-fold higher expression in females^3^. Of note, the differences in fold change observed between our study and previous ones may be attributable to methodological variations and differences in rat strains analysed.

To our knowledge, this is the first genome-wide investigation of sex-specific hepatic gene expressions conducted in Lewis rats. Earlier work has primarily focused on other rat strains such as Fisher^2^ and Sprague-Dawley^3^ rats, as well as various murine models^22–24^. While some RNA-sequencing studies in mice have addressed similar questions, these often concentrate on specific disease states^24^ or experimental treatments^22,23^limiting their generalizability.

Still, we were able to replicate known sex-biased genes, such as *Cyp2c13*^2,3,19^, and due to the increased sensitivity of RNA-sequencing, we also determined novel candidate genes, such as *A1bg*. Importantly, a previous study in rats has demonstrated that these sex-specific expression patterns are also age-dependent, with the most pronounced sex-specific differences detectable between 8 and 52 weeks of age^2^. Although, our study exclusively analysed one timepoint and age, but with approximately 12 weeks of age (86 days), the rats included in our study were within the age range exhibiting the most pronounced sex-specific differences^2^.

For the first time, we provided a comprehensive investigation of differential sex-specific gene expression in individual liver sections. While a substantial number of genes displayed sex-specific expressions in multiple liver sections, our analysis also identified genes exhibiting differences in expression between males and females in an individual liver section. This underscores the potential limitations of analyses conducted on single-sections only in adequately capturing genes with a sex-specific expression. Beyond that, our findings revealed considerable intra-sex heterogeneity between liver sections, with a notably higher number of differentially expressed genes between individual liver sections in male rats. These differences between individual liver sections might have functional implications - particularly given the substantial variations detected. Yet, we cannot determine putative functional consequences based on our data. In fact, this highlights the necessity of rigorous documentation of the liver sections used in future studies, and of conducting further studies to evaluate the biological significance of such spatial heterogeneity. Importantly, sex differences may not only be evident at the transcriptomic level but also at the cellular level. The liver consists of multiple distinct cell types^25^each of which may exhibit sex-specific expression patterns^26,27^further contributing to the observed heterogeneity. Supporting this, a recent single-cell RNA sequencing study in mice found that the majority of sex-biased gene are predominantly found in hepatocytes^23^highlighting the relevance of focussing on specific liver sections or even cell populations to fully understand sex-specific transcriptional regulation.

This heterogeneity may further hold particular relevance for split-liver transplantation. Currently, no data on sex-mismatched outcomes in split-liver transplants has been published, while the debate over whether split-liver transplants yield superior outcomes compared to whole-liver transplants remains ongoing^28–32^. For whole liver transplantation, adverse outcomes in female-to-male transplants are frequently attributed to graft size^33^ which plays a key role in graft allocation decisions^34,35^. However, some studies demonstrated that factors such as donor age and the presence of macrosteatosis have a more pronounced impact on transplantation outcomes than graft size alone^9^. Specifically, the significant association between donor sex and outcomes was no longer detectable when the donor was older than 40 years of age. These findings suggest that graft quality and size may have a lesser effect on post-transplant outcomes compared to other biological factors, such as hormonal differences influenced by donor age or macrosteatosis^9,36^. Thus, particularly given the pronounced sex-specific expression of various genes, a putative impact of the underlying genetics is also feasible.

Our GSEA revealed significant enrichments of male-specific downregulated genes in pathways related to cholesterol homeostasis and oestrogen response. Notably, oestrogen plays a key role in cholesterol regulation, influencing both synthesis and secretion^37^. Well-established sex-specific differences in plasma lipid profiles indicate that females have higher high-density lipoprotein (HDL) and lower levels of low-density lipoprotein (LDL) compared to men^37–39^. Consistently, lipid levels have been shown to dynamically respond to cross-sex hormone therapy in transgender individuals^39^. Supporting oestrogen’s role in cholesterol metabolism, a previous study demonstrated that HMG-CoAR, a key enzyme in cholesterol homeostasis, exhibits lower activity and expression in female mice as well as male mice treated with 17-β-oestradiol^38^. Generally, sex-specific findings in regard to cholesterol homeostasis have broader clinical implications, particularly in context of cardiovascular diseases, where men are predisposed to earlier occurrence of myocardial infarction due to oestrogen’s protective effects on cholesterol homeostasis before menopause^40,41^.

Oestrogen has also been linked to the transplantation outcomes in female-to-male transplants. Studies have shown that oestrogen-related effects may be more relevant than graft size bias in explaining poorer outcomes when a male recipient receives a female organ^9^. Particularly, patients undergoing a female-to-male liver transplantation receiving a graft positive for the oestrogen receptor have been reported to exhibit a higher risk of graft failure within the first six months compared to recipients of negative oestrogen receptor grafts. Of note, this association was specific for female-to-male transplants and has not been observed in other transplantation combinations^9^. Experimental evidence from animal models further support oestrogen’s protective and regenerative role in the liver^42–45^. Female mice have demonstrated greater resistance to liver injury than males, an effect that diminishes following ovariectomy or treatment with oestrogen receptor antagonists, while oestrogen administration in males enhances survival and reduces liver damage. In a rat model of female-to-male liver transplantation, cytosolic oestrogen receptor content in the graft decreased compared to that in normal female livers, but resembling levels similar to male livers. Androgen receptor levels showed the opposite pattern. Interestingly, the ratio between oestrgen receptor and androgen receptor are higher in transplanted female-to-male livers than in unoperated male livers, but lower than in unoperated female livers. This pattern was also seen in male-to-female transplants^46^. Thus, even though the rat livers we have analysed were not subjected to transplantation, the finding that downregulated male-specific genes are enriched for oestrogen related pathway may hint at a putative molecular mechanism rendering transplantation outcomes that needs further investigation on a genetic level.

Nevertheless, caution is warranted when interpreting our GSEA results, as they were derived from human gene sets, and a subsequent GO analysis using a *Rattus norvegicus*-specific database even yielded divergent findings. One potential reason for this discrepancy might be the high proportion of genes (~ 56–62%) that were unambiguously mapped in PANTHER and consequently excluded from the GO analysis. Additionally, unlike the numerous well-annotated and curated human gene sets from MSigDB, annotations for GO analysis are predominantly computationally inferred^47^. Generally, the quantity and quality of human data are significantly superior to those of model organisms like mice and rats^47^.

Yet, our results suggest that gene expression differences, particularly with regard to the detected enrichment of male-specific downregulated genes in pathways such as cholesterol homeostasis, may influence long-term transplantation outcomes. Of note, cholesterol dysregulation has been linked to adverse post-transplant outcomes with one study implying that hypercholesterolemia within one year of living donor liver transplantation is associated with increased risk of graft failure and certain cardiovascular complications^48^.

We are aware of the translation challenges from animal models to human clinical implications. Still, some studies suggest that differences between human and rodent livers are relatively small and that basic structures are equivalent^49,50^. Sex-specific expressions have already been described in the human liver^1,51^. However, many genes with a sex-specific expression in the rat liver are absent in humans. For instance, numerous of the genes we detected to be sex-specifically expressed belong to the cytochrome P450 family, and the number of isoforms varies between humans and rats. *Cyp2c11*, for instance, is not expressed in humans, although an orthologous protein exists for *Cyp2c11* and some other cytochrome P450 genes^52^. Although we did not monitor the menstrual cycle of the female rats, it is rather unlikely that the observed differences in gene expression between the sexes are solely due to this factor, particularly considering that previous studies report inconclusive results regarding its impact on gene expression^53,54^.

Collectively, our study provides a genome-wide coverage of sex-specific gene expressions in livers of Lewis rats. We demonstrated pronounced sex-specific gene expression patterns, with key metabolic pathways such as cholesterol homeostasis and oestrogen response being significantly affected. Further, we provided novel evidence of inter- and intra-sex heterogeneity in gene expression across individual liver sections, uncovering putative limitations and biases of single-section analyses. Future studies should explore functional implications of these gene expression differences in a transplantation setting, particularly focussing on sex-mismatched donor-recipient pairs.

## Materials and methods

### Ethics statement

All animal procedures were conducted in accordance with the ARRIVE guidelines and adhered to relevant institutional, national, and European regulations governing the ethical use of animals in research, including EU Directive 2010/63/EU and the German Animal Welfare Act (Tierschutzgesetz). Ethical approval for all procedures was granted by the Lower Saxony State Office for Consumer Protection and Food Safety (LAVES), Oldenburg, Germany, under protocol number 2018/623.

### Study design

Liver extraction was performed in two groups based on the sex of the rats. Thus, four male and four female animals were included to enable downstream analysis of sex-specific gene expression profiles.

### Animals, housing and husbandry

A total of eight Lewis rats (Lew/NHanZtm) were included in the study, consisting of four males and four females. All animals were 86 days old (~ 12 weeks) at the time of organ harvest. Average body weights were approximately 300 g for males and 200 g for females. The rats were obtained from the central animal facility of Hannover Medical School, where they were housed under standardized conditions in accordance with EU and German guidelines, including regulated temperature, humidity, and *ad libitum* access to food and water. The rats were maintained on a 14-hour light / 10-hour dark cycle with lights on at 6:00 AM (Zeitgeber time (ZT) 0) and lights off at 8:00 PM (ZT 14). Hepatectomy was performed between 9:00 AM and 11:00 AM, corresponding to ZT 3 to ZT 5.

### Euthanasia

Animals were monitored daily, and no adverse events or clinical signs of distress were observed prior to euthanasia. Euthanasia was performed by gradual-fill exposure to carbon dioxide (CO₂) at a flow rate of 3–7 L/min in a transparent chamber with a volume of approximately 10 L, supplemented by ambient air. Flow was maintained for at least one minute after the cessation of respiratory movements and the onset of ocular pallor, in line with AVMA guidelines^55^.

### Tissue collection

**Fig. 7:**
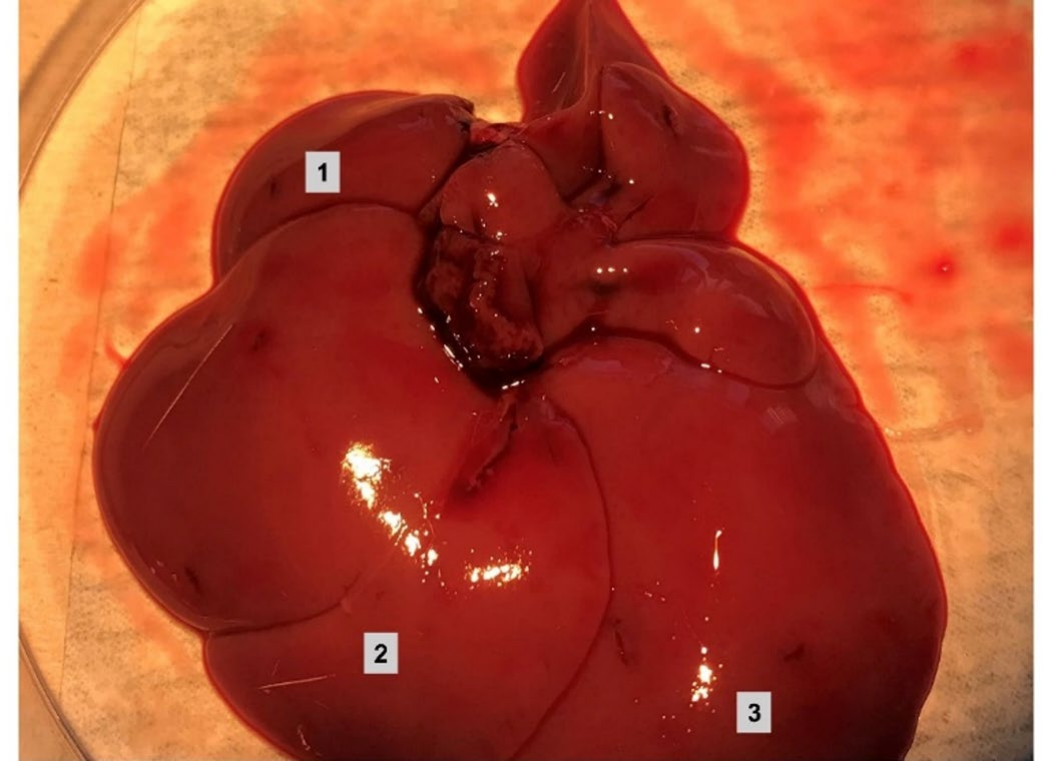
Analysed individual sections of rat livers. Each liver of the included female (*n* = 4) and male (*n* = 4) Lewis rats was sectioned into the three shown individual parts. Each section was separately stored and eventually sequenced. Section 1 represents the right lateral lobe; Section 2 corresponds to the median lobe and Section 3 refers to the left lateral lobe.

Immediately following euthanasia, animals underwent median laparotomy. Exsanguination was achieved by transection of the inferior vena cava and abdominal aorta. The livers were carefully explanted, segmented into three predefined anatomical sections (see Fig. 7; Sect. 1: right lateral lobe, Sect. 2: median lobe and Sect. 3: left lateral lobe), snap-frozen in liquid nitrogen, and stored at − 80 °C for downstream analysis.

### Experimental outcomes

Gene expression was analysed across all liver sections between males and females. Further, sex-specific gene expression within each section was analysed individually. Analyses of differences in gene expressions between the sections within each sex ensued.

### RNA isolation

To ensure successful RNA isolation and sufficient yield, all liver sections (three sections per rat) were weighed and stored in a mix of lysis buffer RA1 (Macherey Nagel, Düren, Germany) containing the reducing agent 2-mercaptoethanol. For each 30 mg of liver tissue, 350 µl of lysis buffer RA1 and 3.5 µl of 2-mercaptoethanol were added. The liver tissue was subsequently minced using a scalpel followed by mechanical disruption. RNA was isolated from the disrupted liver sections using the NucleoSpin RNA kit (Macherey Nagel, Düren, Germany) according to the manufacturer’s manual. Isolated total RNA was subsequently stored at -80 °C.

RNA quality and quantity was assessed using the NanoDrop 2000 (ThermoScientific, Waltham, MA, USA) and the Agilent Bioanalyzer (Santa Clara, CA, USA) to ensure sufficient quality for subsequent RNA sequencing. All samples (*n* = 24, three liver sections from each rat) exhibited an RNA Integrity Number (RIN) of at least 7.4. The RNA quality was further checked with the Qubit (Invitrogen, Waltham, MA, USA) and an RNA bleach gel^56^. To evaluate the DNA digestion which was part of the NucleoSpin RNA kit, a semi-quantitative polymerase chain reaction (PCR) with a subsequent 2.5% agarose gel electrophoresis was performed.

### RNA sequencing and bioinformatic analyses

Library preparation was performed with QuantSeq 3’ mRNA-Seq Library Prep kit FWD (Lexogen, Vienna, Austria). RNA sequencing was subsequently performed on a NextSeq2000 (Illumina, San Diego, CA, USA). The raw data can be accessed in the Sequence Read Archive (PRJNA1258763). Raw sequences were trimmed with TrimGalore (v.0.6.0)^57^ and aligned to the *Rattus norvegicus* genome (rn6) using hisat2^58^.

Statistical analyses were performed with R (v.4.2.0)^59^. Transcript IDs were matched to the respective gene symbols using the R-package *org.Rn.eg.db* (version 3.18.0). Genes which were expressed in exclusively one sample as well as genes with a mean expression below five were excluded from subsequent analyses (see Table 1).

**Table 1:** Number of genes included in the differential gene expression analyses.

Comparison	Number of genes included in analyses after filtering
All male vs. all female sections	11,479
Male right lateral lobe vs. female right lateral lobe	12,479
Male median lobe vs. female median lobe	12,299
Male left lateral lobe vs. female left lateral lobe	12,070
Female right lateral lobe vs. female median lobe	12,695
Female right lateral lobe vs. female left lateral lobe	12,571
Female median lobe vs. female left lateral lobe	12,552
Male right lateral lobe vs. Male median lobe	12,323
Male right lateral lobe vs. Male left lateral lobe	12,126
Male median lobe vs. Male left lateral lobe	12,017

In total, 32,883 transcripts were detected by RNA-sequencing. Prior to the differential gene expression analyses, genes were filtered. Consequently, only genes expressed in more than one sample as well as genes with a mean expression of at least five remained in the analyses.

Differential gene expression (DGE) analysis was performed using DESeq2 (with two-sided Wald test)^60^. For sex-specific expression analyses, such as the comparison between female right lateral lobe versus male right lateral lobe, the variable ‘sex’ was included in the DESeq2 design formula. For section-specific expression analyses within one sex, like the comparison of female right lateral lobe versus female median lobe, the variable ‘individual’ was added. UMAP was performed using the R package ‘umap’ (v0.2.8.0)^61^. To determine putative functional enrichments of the identified differentially expressed genes (p_adj_ < 0.05), GSEA was conducted using the R package ‘fgsea’ (v1.28.0)^15^. Initially, human homologues of rat genes were investigated^14^in order to ensure the usage of well annotated gene set data. The annotated gene sets ‘Hallmark’^62^the curated gene set ‘C2’, the regulatory target gene set ‘C3’, the ontology gene set ‘C5’ as well as the cell type signature gene set ‘C8’ from the ‘Molecular Signatures Database’^13,14^ (downloaded on 14. January 2025 from https://www.gsea-msigdb.org/gsea/msigdb/index.jsp) were analysed. To further explore overrepresentation in processes and pathways related to *Rattus norvegicus*, a GO enrichment analysis was performed applying the PANTHER overrepresentation test with (Fisher’s exact test with Bonferroni correction; version 2024-11-03)^16^. The ‘GO biological processes’, ‘GO molecular functions’ and ‘GO cellular component’ annotation datasets were analysed.

The results of the DGE analyses were visualized using the R packages ‘EnhancedVolcano’ (v1.20.0)^63^‘ggplot2’ (v3.5.1)^64^ and ‘ComplexHeatmap’ (v2.18.0)^65,66^.

## Supplementary Information

Below is the link to the electronic supplementary material.

Supplementary Material 1

Supplementary Material 2

## Data Availability

The datasets generated and analysed during the current study are available in the Sequence Read Archive repository under the accession number: PRJNA1258763.
