# Supplementary material for: Distinct liver sections exhibit sex-specific gene expression patterns in Lewis rats
Source: Sci Rep. 2025 Sep 1;15:32143. doi: 10.1038/s41598-025-17729-0 (PMC12402114; doi:10.1038/s41598-025-17729-0)
Supplement: Supplementary file 1 — Supplementary Material 1 [file 41598_2025_17729_MOESM1_ESM.pdf]

Supplementary Figures for "Distinct liver sections exhibit sex-specific gene expression patterns in Lewis rats" by Rajcsanyi et al.

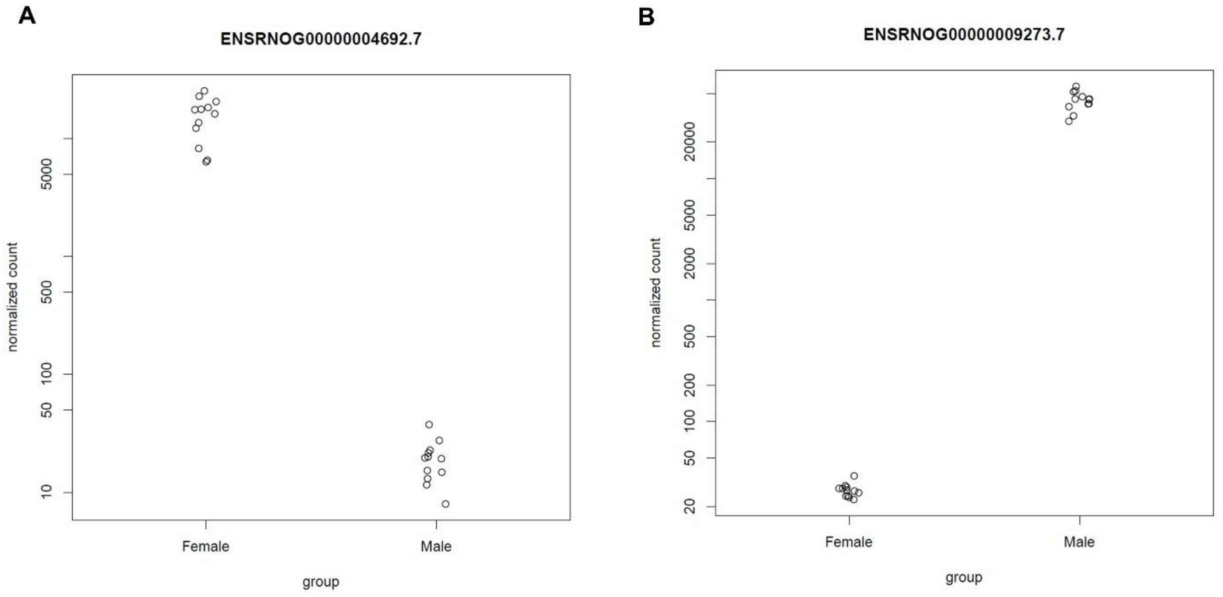

Supplementary Figure 1: Normalized counts of the most differentially expressed genes in males compared to females across all liver sections. The most downregulated (A) or upregulated (B) genes in the comparison of male and female rat liver across all individual sections. Each point represents an individual sample.

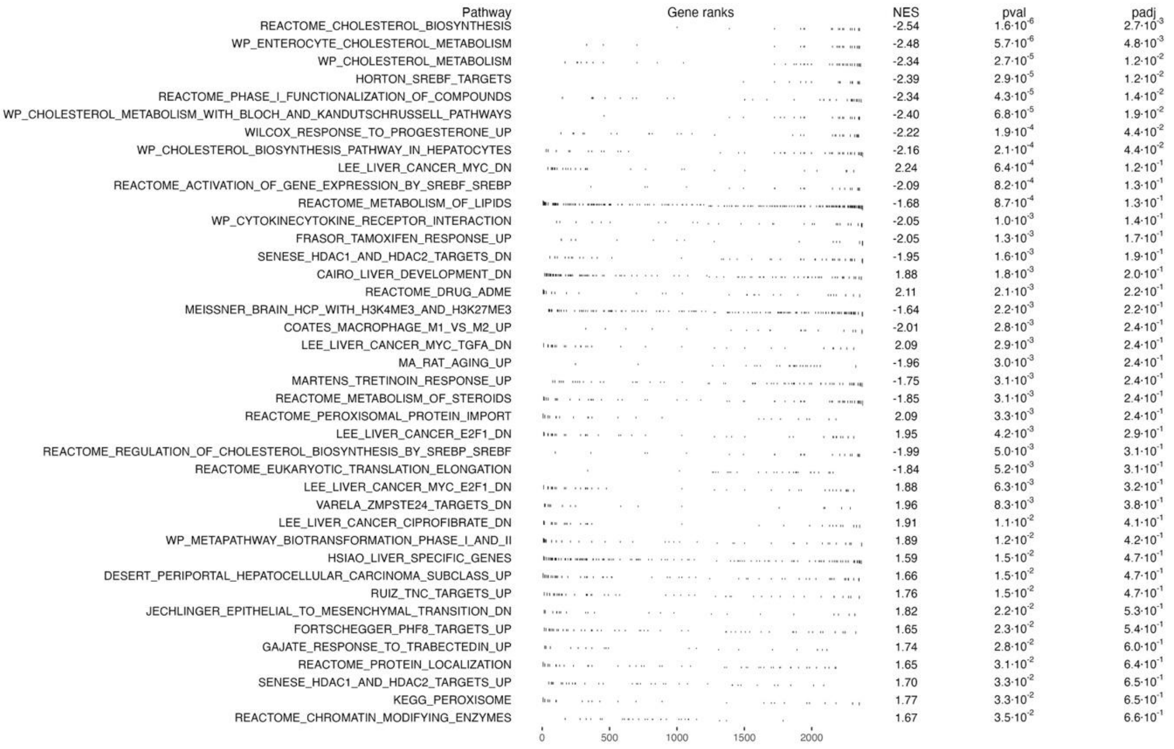

Supplementary Figure 2: GSEA using the human curated gene set C2 across all liver sections of male and female rats. GSEA was performed with the curated gene set C2 to identify pathways enriched in differentially expressed genes between female and male rats. The most significantly enriched pathways are presented ranked according to their p-value. A positive normalized enrichment score (NES) indicates pathways with an enrichment of genes upregulated in males, while negative NES imply to pathway enrichments of genes downregulated in males. Pathway enrichment was considered significant if $p_{adj} < 0.05$.

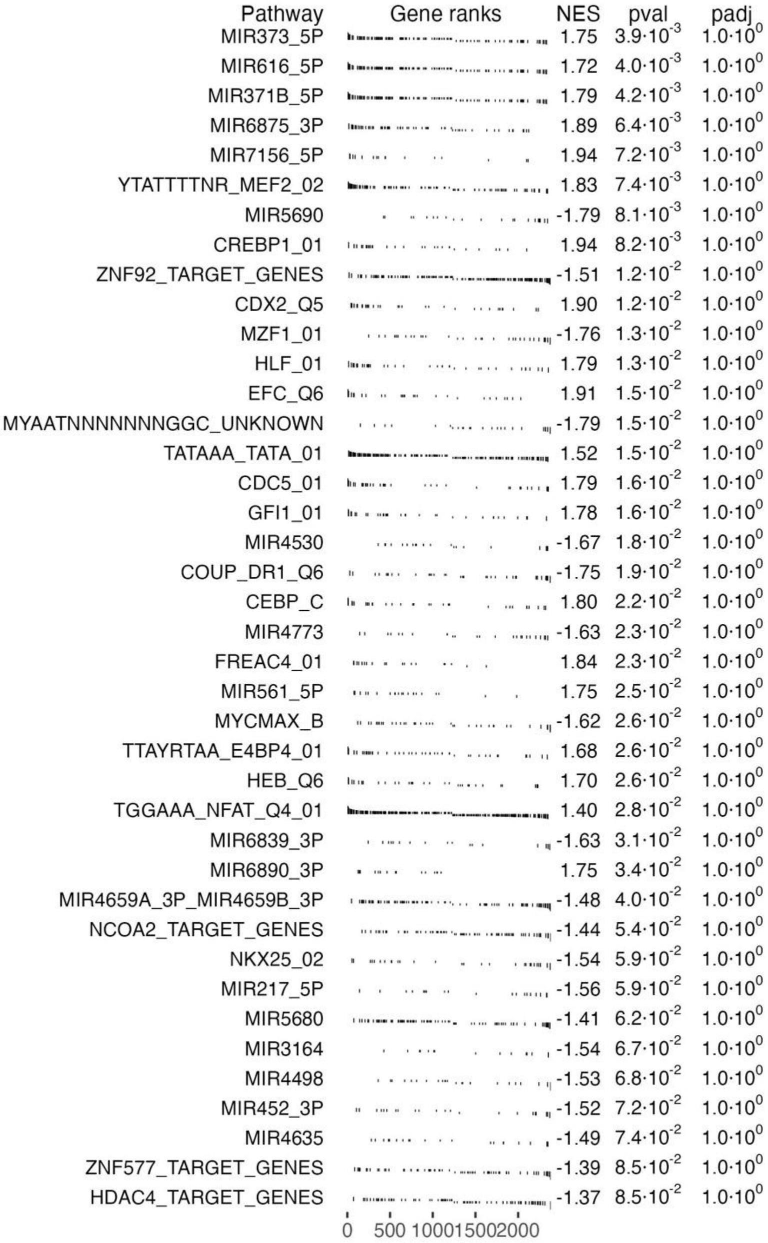

Supplementary Figure 3: GSEA using the human regulatory gene set C3 across all liver sections of male and female rats. GSEA was performed with the regulatory gene set C3 to identify pathways enriched in differentially expressed genes between female and male rats. The most significantly enriched pathways are presented ranked according to their p-value. A positive normalized enrichment score (NES) indicates pathways with an enrichment of genes upregulated in males, while negative NES imply to pathway enrichments of genes downregulated in males. Pathway enrichment was considered significant if $p_{adj} < 0.05$.

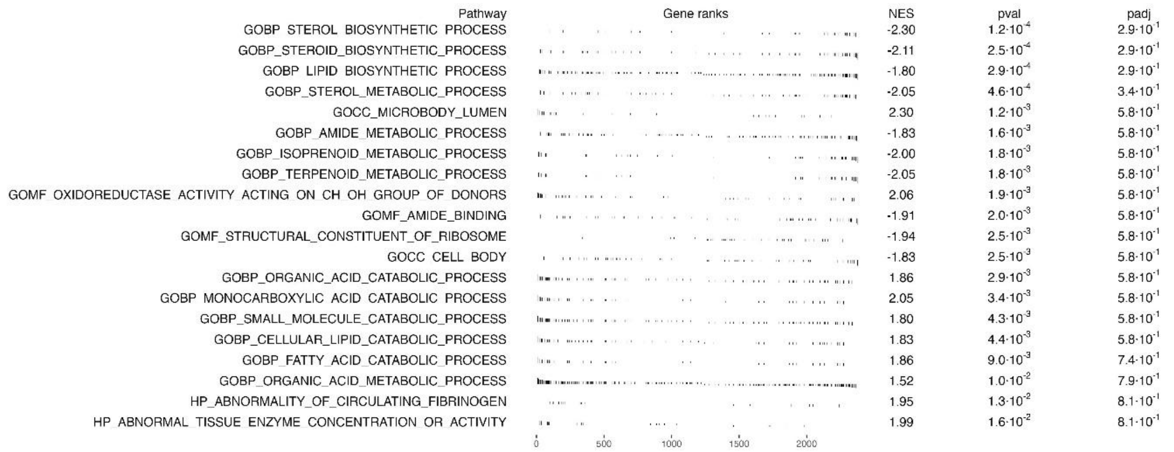

Supplementary Figure 4: GSEA using the human ontology gene set C5 across all liver sections of male and female rats. GSEA was performed with the ontology gene set C5 to identify pathways enriched in differentially expressed genes between female and male rats. The most significantly enriched pathways are presented ranked according to their p-value. A positive normalized enrichment score (NES) indicates pathways with an enrichment of genes upregulated in males, while negative NES imply to pathway enrichments of genes downregulated in males. Pathway enrichment was considered significant if $p_{adj} < 0.05$.

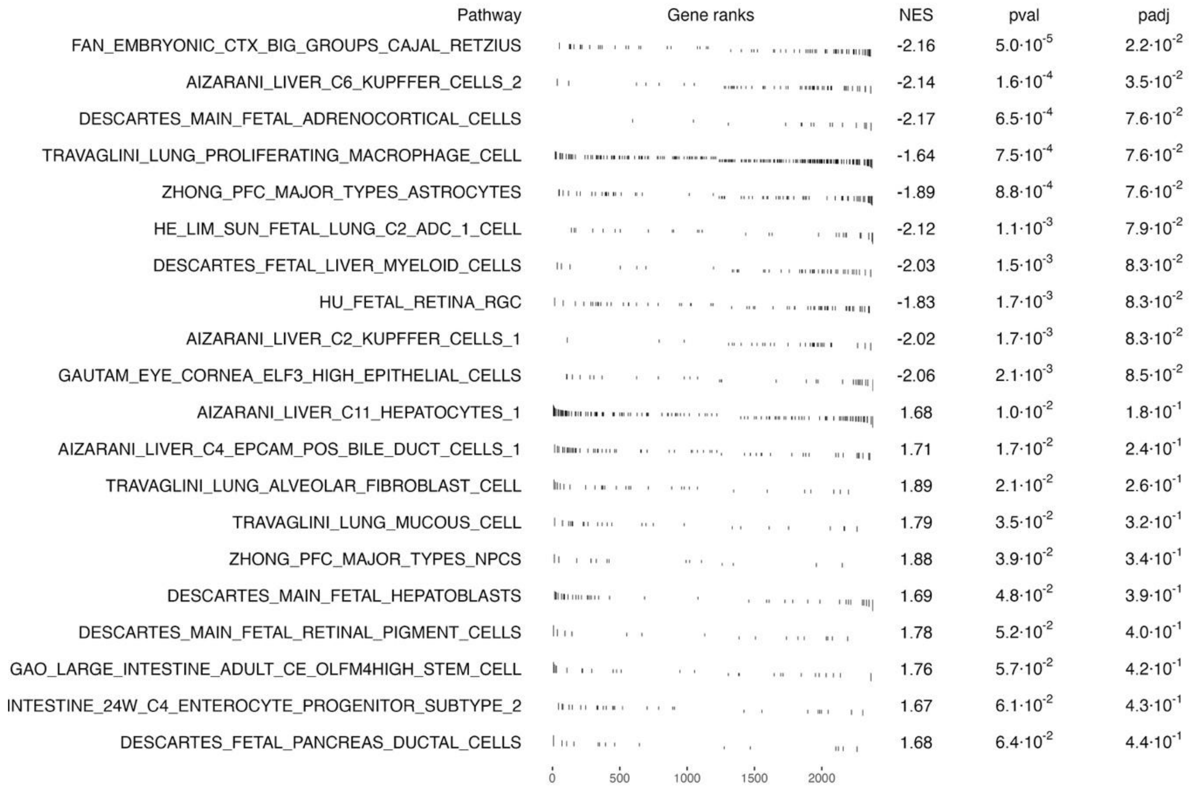

Supplementary Figure 5: GSEA using the human cell type signature gene set C8 across all liver sections of male and female rats. GSEA was performed with the cell type signature gene set C8 to identify pathways enriched in differentially expressed genes between female and male rats. The most significantly enriched pathways are presented ranked according to their p-value. A positive normalized enrichment score (NES) indicates pathways with an enrichment of genes upregulated in males, while negative NES imply to pathway enrichments of genes downregulated in males. Pathway enrichment was considered significant if $p_{adj} < 0.05$.

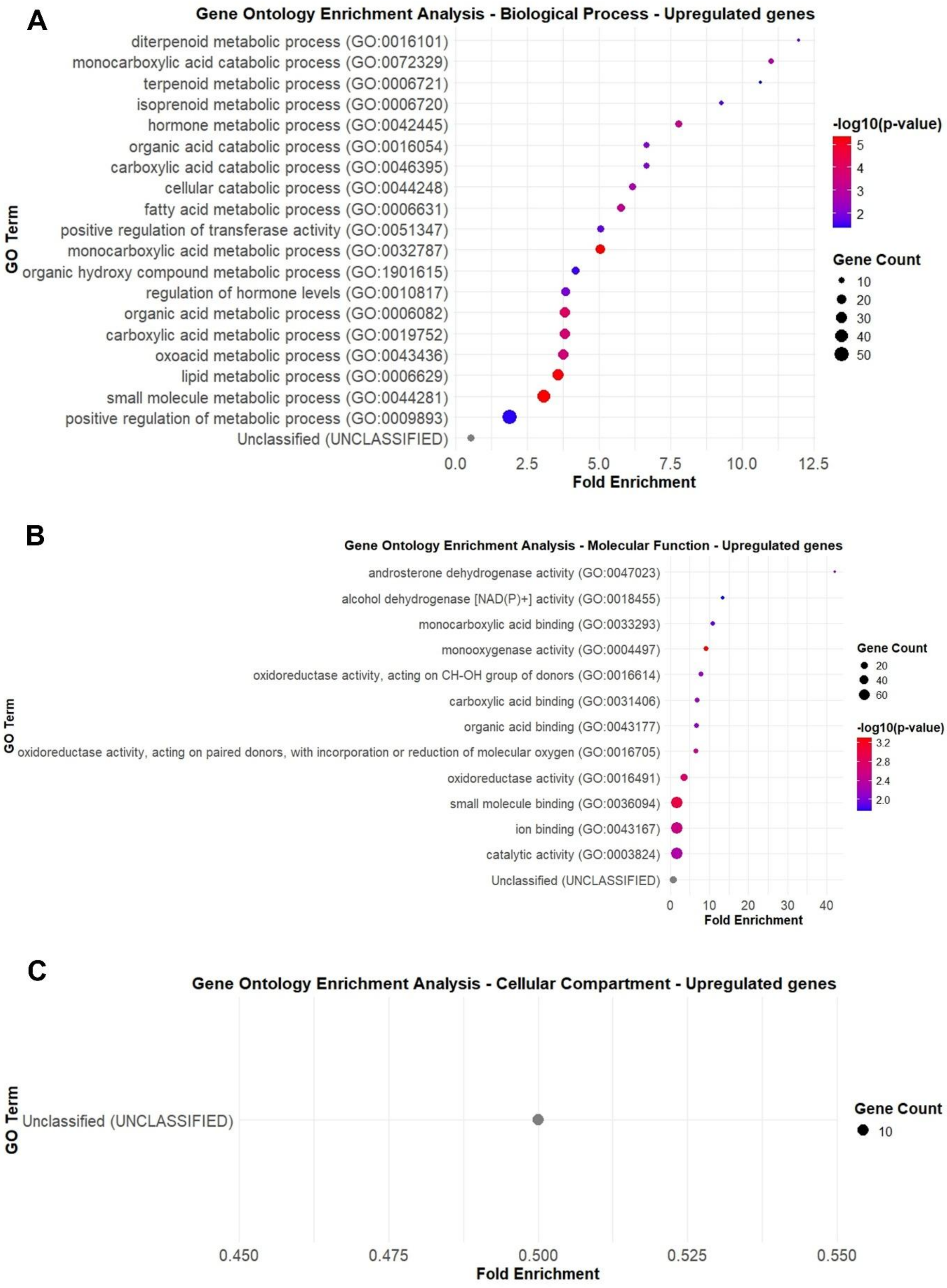

Supplementary Figure 6: GO enrichment analysis of genes significantly upregulated in males compared to female rats across all liver sections. Here, only gene significantly upregulated in males compared to females ($p_{adj} < 0.05$, $\log_2 FC > 1$) were included in the analyses. For the analysed annotation datasets *GO biological process* (A), *GO molecular function* (B) and *GO cellular component* (C), the logarithmic p-value is indicated by the color, while the corresponding gene count detected in the uploaded gene list is further represented by the dot size. GO: Gene Ontology

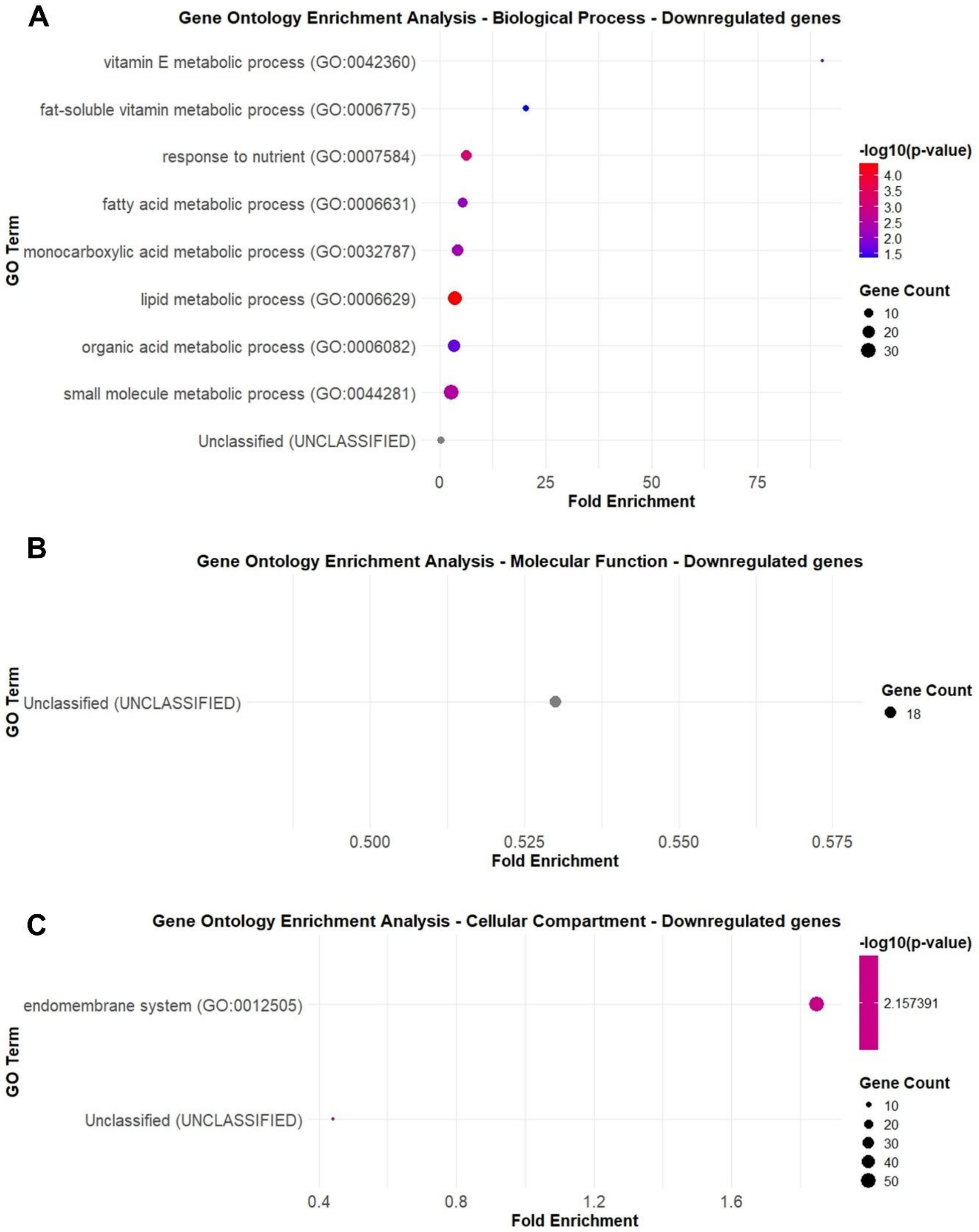

Supplementary Figure 7: GO enrichment analysis of genes significantly downregulated in male compared to female rat across all liver sections. Here, only genes significantly downregulated in males compared to females ($p_{adj} < 0.05$, log2FC < -1) were included in the analyses. For the analysed annotation data sets GO Biological Process (A), GO Molecular Function (B) and GO Cellular Component (C), the logarithmic p-value is indicated by the color, while the corresponding gene count detected in the uploaded gene list is further represented by the dot size. GO: Gene Ontology.

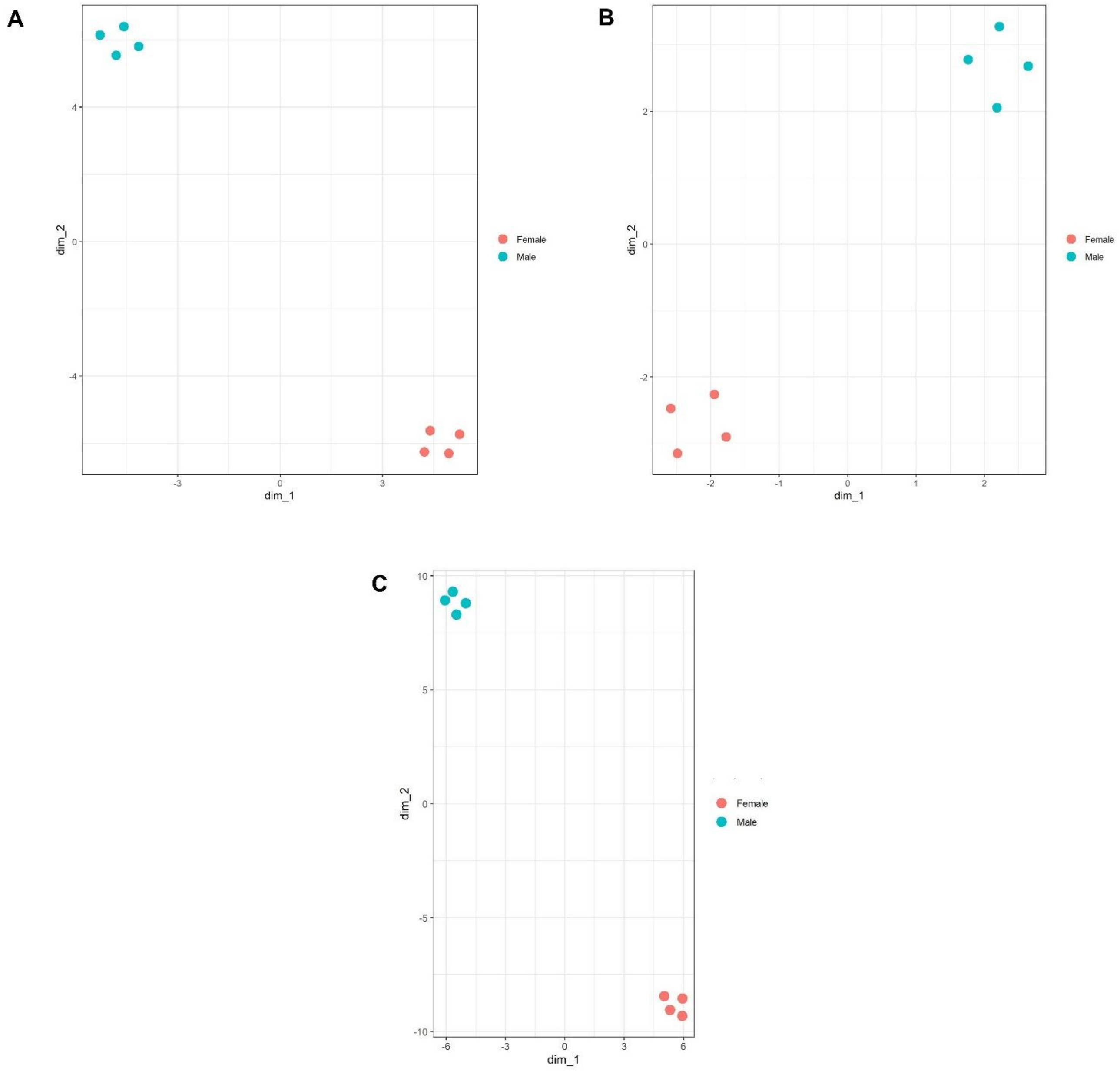

Supplementary Figure 8: UMAP of section-specific gene expression analyses in male and female rats. The UMAP of samples obtained from female (n = 4) and male (n = 4) right lateral lobe (A), median lobe (B) and left lateral lobe (C) are shown. The corresponding segmentation of each liver is illustrated in Figure 7. Red dots indicate samples from female rat livers, while blue dots represent male liver samples.

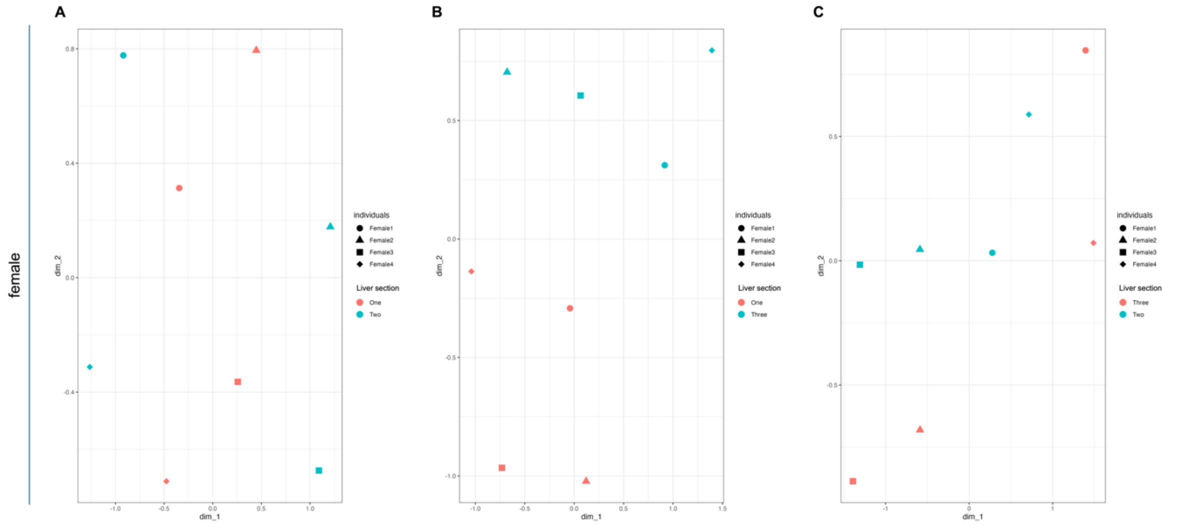

Supplementary Figure 9: UMAP of liver section comparison within female rats. Samples of female (n = 4) Lewis rats were sectioned into three individual parts: right lateral lobe, median lobe, and left lateral lobe. Here, comparison of two individual sections each in one sex is shown: A) female right lateral lobe versus female median lobe, B) female right lateral lobe versus female left lateral lobe, C) female median lobe versus left lateral lobe. The corresponding segmentation of each liver is illustrated in Figure 7. The individual rats are represented with different symbols, while the analysed liver sections are indicated by the colours.

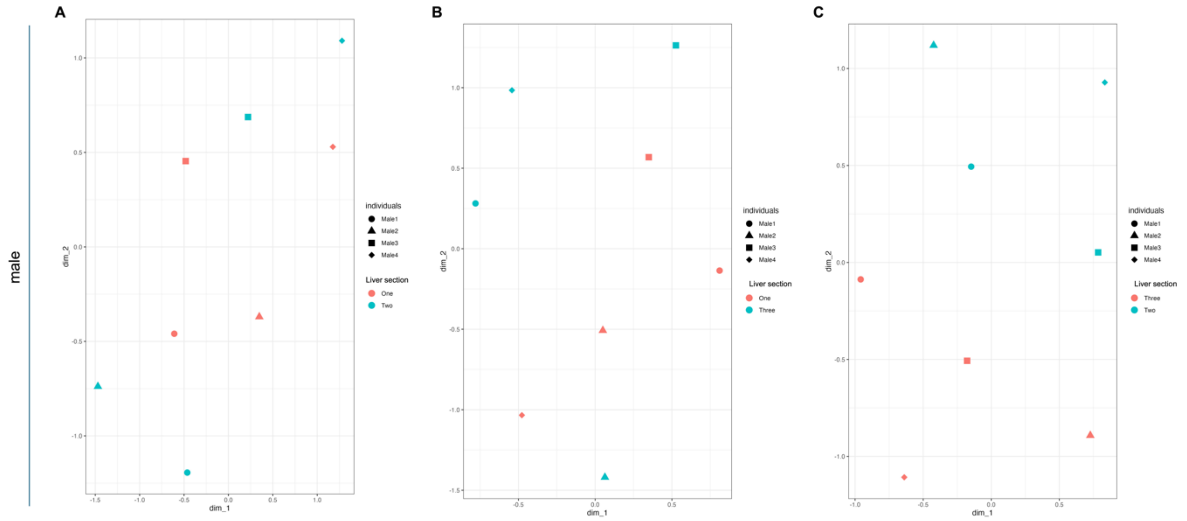

Supplementary Figure 10: UMAP of liver section comparison within male rats. Samples of male (n = 4) Lewis rats were sectioned into three individual parts: right lateral lobe, median lobe, and left lateral lobe. Here, comparison of two individual sections each in one sex is shown: A) male right lateral lobe versus male media lobe, B) male right lateral lobe versus male left lateral lobe, C) male median lobe versus male left lateral lobe, The corresponding segmentation of each liver is illustrated in Figure 7. The individual rats are represented with different symbols, while the analysed liver sections are indicated by the colours.
